# Proposal for the SOSORT inclusion criteria for studies on physiotherapy

**DOI:** 10.1186/1748-7161-7-S1-O54

**Published:** 2012-01-27

**Authors:** HR Weiss, E Santos Leal, U Hammelbeck

**Affiliations:** 1 Orthopedic Rehabilitation Services, Gensingen, Germany

## Background

Studies investigating the outcome of conservative scoliosis treatment differ widely with respect to the inclusion criteria used [1]. While the application of the SRS criteria for studies on bracing seem useful, there are no inclusion criteria for the investigation of physiotherapy alone. This study has been performed to investigate the possibility of useful inclusion criteria for future prospective studies on physiotherapy (PT).

## Materials and methods

A PubMed and (incomplete) hand search for outcome papers on PT has been performed in order to detect study designs and inclusion criteria used.

## Results

Real outcome papers (start of treatment in immature samples / end results after the end of growth) have not been found. Some papers investigated mid-term effects of exercises, most were retrospective, few prospective [2] and some included patient samples with questionable treatment indications [3].

## Discussion

An agreement of the scientific community on common inclusion criteria for future studies on PT is necessary. We would suggest the following: (1) girls only, (2) age 10 to 13 with the first signs of maturation (Tanner II), (3) Risser 0-2, (4) risk for progression 40 – 60% according to Lonstein and Carlson.

## Conclusions

A SOSORT consensus paper following a 3-step Delphi process seems necessary in order to establish the inclusion criteria for future studies on PT.
